# Bio particle swarm optimization and reinforcement learning algorithm for path planning of automated guided vehicles in dynamic industrial environments

**DOI:** 10.1038/s41598-024-84821-2

**Published:** 2025-01-02

**Authors:** Shiwei Lin, Jianguo Wang, Bomin Huang, Xiaoying Kong, Hongwu Yang

**Affiliations:** 1 School of Computer Engineering, Jimei University, Xiamen, 361000 Fujian China; 2 Faculty of Engineering and Information Technology, University of Technology Sydney, Sydney, 2007 NSW Australia; 3 School of IT and Engineering, Melbourne Institute of Technology (Sydney Campus), Sydney, 2000 NSW Australia; 4 Xiamen Topstar Co., Ltd., Xiamen, 361000 Fujian China

**Keywords:** Computational science, Mechanical engineering

## Abstract

Automated guided vehicles play a crucial role in transportation and industrial environments. This paper presents a proposed Bio Particle Swarm Optimization (BPSO) algorithm for global path planning. The BPSO algorithm modifies the equation to update the particles’ velocity using the randomly generated angles, which enhances the algorithm’s searchability and avoids premature convergence. It is compared with Particle Swarm Optimization (PSO), Genetic Algorithm (GA), and Transit Search (TS) algorithms by benchmark functions. It has great performance in unimodal optimization problems, and it gains the best fitness value with fewer iterations and average runtime than other algorithms. The Q-learning method is implemented for local path planning to avoid moving obstacles and combines with the proposed BPSO for the safe operations of automated guided vehicles. The presented BPSO-RL algorithm combines the advantages of the swarm intelligence algorithm and the Q-learning method, which can generate the globally optimal path with fast computational speed and support in dealing with dynamic scenarios. It is validated through computational experiments with moving obstacles and compared with the PSO algorithm for AGV path planning.

## Introduction

Automated Guided Vehicle (AGV) achieves high flexibility, efficient and economical unmanned production in the logistics system, material transportation, and port environment^1,2^. AGV solves the problems of task scheduling and path planning in the intelligent manufacturing workshop^3^. Navigation of autonomous robots includes three general problems: path planning, localization and motion control. Path planning involves following an optimal path without colliding with obstacles^4^.

Path planning is referred to as an NP-hard problem in optimization and considers substantial optimality criteria, such as safety, smoothness, operation time, and path length^5^. Regarding implementation, path planning algorithms can be divided into real-time and offline implementation^5^. According to the information on the work area, AGV path planning is divided into global and local path planning. Global path planning refers to complete regional information, while local path planning requires real-time performance based on local environmental information^1^.

This paper proposes an improved path-planning algorithm based on the swarm intelligence algorithm and implements reinforcement learning for local path planning to avoid moving obstacles. It provides faster computational speed to gain the best fitness value when compared with other swarm intelligence algorithms, and it is suitable for AGV path planning to generate the optimal path. The paper’s main contributions are as follows:

- An improved swarm intelligence algorithm based on Particle Swarm Optimizaiton (PSO) is proposed as the Bio PSO (BPSO) algorithm, which modifies the updating equation for particles’ velocity.
- The proposed BPSO algorithm is used for global path planning to generate the optimal path in the industrial environments.
- It integrates the proposed algorithm with the Q-learning method for local path planning for AGVs’ autonomous operations.

This paper is organized as follows. Section Related work presents the related work of AGV path planning, and Section Problem statement formulates the path planning problem. Section Bio particleswarm optimization-reinforcement learning (BPSO-RL) describes the proposed BPSO-RL algorithm for global and local path planning, and the experiment results are shown in Section Experiments. It is concluded in Section Conclusion.

## Related work

Geometric search algorithms are classical path planning algorithms, such as Dijkstra algorithm^6,7^, A* algorithm^2,8,9^, D* Lite algorithm^10^. Also, other classical path planning algorithms include rapidly exploring random trees (RRT)^8,11^, Probabilistic road map (PRM)^12^, and Artificial Potential Field (APF)^13^. An improved A* algorithm is introduced for AGV by setting the filter function to reduce the turning angles and combining it with the cubic B-spline interpolation function for continuous speed and acceleration^2^. Reference^14^ considers collision risk and path length for the optimal path as an improved A* algorithm with a multi-scale raster map and combines the Line-of-sight algorithm.

Reference^8^ combined A* and RRT algorithms to improve search efficiency and reduce path conflicts for multi-AGV routing. Kinematical constraint A* is integrated with the Dynamic window algorithm (DWA) for dynamic AGV path planning to reduce the number of turns and the length and time of the path^9^. APF is combined with a deep deterministic policy gradient framework for the port environment to guarantee the safety and smoothness of the path^15^.

Intelligent bioinspired algorithms are widely used in AGV path planning, such as PSO^3^, Genetic Algorithm (GA)^16^, and Ant Colony Optimization (ACO)^17^ algorithms, etc.^3^ proposed an improved PSO (IPSO) algorithm with a new coding method and a crossover operation in for AGV in material transportation, adopting a mutation mechanism to prevent falling into the local optimum.

Reference^18^ designed a hybrid evolutionary algorithm based on PSO to avoid trapping in the local optima by updating the inertia weight based on a probabilistic approach in real-time implementation with a dual-layer framework, achieving collision avoidance, fault tolerance, and task allocation for multi-AGV path planning. The improved GA has three-exchange crossover operators and double-path constraints to minimize the path distance for multi-AGV path planning^16^. An improved Global Dynamic Evolution Snow Ablation Optimizer is designed to solve global optimization and path planning problems, which uses a dynamic snowmelt ratio and a neighbourhood dimensional search scheme^19^.

To overcome the shortcomings of ACO in weak optimization ability and slow convergence for global path planning, an improved ACO uses fruit fly optimization (FOA) for pre-searching to obtain the pheromone distribution, then using ACO for global path planning^17^. A parallel ACO is presented with a multi-objective function that includes the number of turns and the shortest path through the interaction of pheromones, and it improves the working and processing efficiency in the warehouse^20^.

A binary PSO with velocity control is introduced in Ref.^21^, which is a modified version of the PSO algorithm with two velocity vectors for each particle. A variable velocity strategy PSO is presented with adding a new term in the velocity updating process that is controlled by a reduction linear function as a novel movement strategy^22^. PSO is integrated with social group optimization to propose a velocity adaptation algorithm for localization problems, which considers average velocity and partial derivative of personal and global best values^23^.

Reference^24^ presents a modified heat transfer search algorithm based on the heat transfer search algorithm and sub-population-based simultaneous heat transfer mode to improve the population diversity and effectiveness. Reference^25^ designed a modified teaching-learning-based optimization by introducing self-motivated learning, multiple teachers, adaptive teaching factor and learning through tutorials. Reference^26^ formulates the aerodynamic model of a small fixed-wing Unmanned Aerial Vehicle (UAV) into sub-systems, and it compares 13 metaheuristics approaches for the proposed system identification optimization problem.

Additionally, reinforcement learning has attracted attention to solving the problem of AGV path planning. An improved Q-learning path optimization is proposed for dynamic working stations based on Kohonen networks and an enhanced GA for local and global path planning, respectively^27^. Asynchronous Advantage Actor-critic (A3C) is combined with attention mechanism in the storage multi-pick station, resulting in increased reward and faster convergence^28^.

Reference^29^ used the Dueling Double Deep Q Network to learn the AGVs’ control with prioritized experience reply for intelligent logistics systems and using multi-modal sensors to avoid obstacles and reach the target. Reference^30^ designed a remote path planning approach based on ACO and reinforcement learning to reduce the blindness of ACO searching, and the path generated by ACO is used for training, then selecting the optimal action based on the Q table.

An integrated framework implements a bootstrapped deep Q-Network for adaptive decision-making of autonomous vehicles and achieves path planning by an inverse RL approach^31^. Reference^32^ presents a twisted Gaussian risk model for host vehicle trajectory planning, and^33^ uses a lane crossing and final points generation model-based trajectory prediction approach based on long short-term memory and deep conditional generative model. Reference^34^ introduces a type-3 fuzzy controller for path-tracking, and it is under the assumption of unknown and non-linear system dynamics.

Reference^35^ designs a vehicle-to-vehicle (V2V) communication by an intersection-based distributed routing strategy and uses ACO for optimal path; another autoregressive integrated moving average model for the V2V routing is presented in Ref.^36^. Reference^37^ proposes a bus-trajectory-based street-centric routing algorithm for message delivery and uses ACO for a bus-based forward strategy. Reference^38^ uses an inertial-aided Unmodulated Visible Light Positioning system for pedestrian navigation, and it implements smartphones for precise ranging and tightly coupled integration in an optimization-based framework.

Reference^39^ develops a human-like trajectory planning model using the driver preview mechanism with a data-driven method. A cascade attention mechanism is presented to enhance the performance of road traffic sign recognition, and it designs a mutual attention enhancement module, modal fusion mechanism, and a deep learning model^40^. Reference^41^ designs an intersection energy consumption and emissions model framework to evaluate different signal priority strategies.

However, classical algorithms may waste their available space. The intelligent bioinspired and reinforcement learning algorithms have gained attention in AGV path planning. The intelligent bioinspired algorithms effectively generate global paths, but they suffer from limited adaptability for moving obstacles or being trapped in the local optima. The reinforcement learning would result in slow convergence in large spaces, but it is suitable for dealing with dynamic scenarios. The paper combines the advantages of bioinspired and reinforcement learning algorithms to perform global and local path planning to avoid moving obstacles with enhanced computational speed and the ability of exploration and exploitation. The presented BPSO-RL algorithm proposes a new velocity update strategy, and it introduces a random variable to enhance the searchability in the solution space to avoid premature convergence, and it is implemented in path planning for AGVs.

## Problem statement

AGV path planning aims to find the optimal path from the start to the target location without collisions. The industrial environment is modelled as the grid map, and the map is represented by binary numbers: 0 for the free space and 1 for the obstacles or the walls. The start location is represented by $S(x_{start},y_{start}) \in {\mathbb {R}}^2$, and the target location is represented by $T(x_{target},y_{target}) \in {\mathbb {R}}^2$. The path P consists of path points $\left\{ p_1,p_2,\cdots ,p_n \right\}$, and the coordinate of the path point $p_{n,t} \in P$ is $(x_n,y_n)$ at timestamp *t*. The obstacles are set as $O_i (x_{o,i},y_{o,i},{info}_{o,i})$, and ${info}_{o,i}$ records additional information about the current obstacle $O_i$. The objective function of AGV path planning for BPSO is formulated as Eq. (1).

1 $$\begin{aligned} \begin{aligned} \mathop {\textrm{minimize}}_{(x_{k+1},y_{k+1})\in {\mathbb {R}}^{2}}~~&f_{path}(x_{k+1},y_{k+1})= w_1 \cdot f_{length}+ w_2 \cdot f_{collision}\\ \mathrm {subject~to}~~&(x_{k+1},y_{k+1})~~\ne ~(x_{k},y_{k}) \end{aligned} \end{aligned}$$

where $(x_{k}, y_{k})$ is the coordinate of the AGV in the iteration *k*. $w_1$ and $w_2$ are the weight factors for each objective function, and their sum is 1. Eqs.(2)–(5) is to minimize the path length and avoid collisions.

2 $$\begin{aligned} d (p_{k+1}^t, p_k^t)= &   \sqrt{(x_{k+1}^{t}-x_{k}^{t})^{2} + (y_{k+1}^{t}-y_{k}^{t})^{2}} \end{aligned}$$

3 $$\begin{aligned} f_{length}^t= &   \sum _{k=1}^{n-1} d (p_{k+1}^t, p_k^t) \end{aligned}$$

4 $$\begin{aligned} c_k^t= &   \sum _{c=1}^{j} r_c - d_{c}^t, ~if~d_{c}^t <r_c \end{aligned}$$

5 $$\begin{aligned} f_{collision}^t= &   \sum _{k=1}^{n} c_k^t \end{aligned}$$

where $$d (p_{k+1}^t, p_k^t) $$ is the distance between path points $$p_{k+1}^t$$ and $$p_k^t$$ in the current timeslot *t*. $$c_k^t$$ is the value of collied obstacles, $$d_c^t$$ is the current distance from the particle to the centre of the obstacle, and $$r_c$$ is the radius of the obstacle.

The reward function for the QL method is defined as Eq. (6).

6 $$\begin{aligned} R(s, a) = {\left\{ \begin{array}{ll} -R_{collision} &  \text {if } s_{k+1} \in O_i, \\ R_{goal} &  \text {if } s_{k+1} = T(x_{target},y_{target}), \\ -R_{moving} \end{array}\right. } \end{aligned}$$

where $R_{collision}$ is the penalty if the next state $s_{k+1}$ is collided with the obstacle, $R_{goal}$ is the reward for reaching the goal, and $R_{moving}$ is the moving reward.

The assumptions are as follows:

- AGVs are assumed to be moved at a constant speed within the environment
- AGVs can be communicated immediately
- AGVs support multi-angle steering
- The proposed BPSO-RL algorithm is implemented in the AGVs’ board
- AGVs are aware of the environment, and they carry sensors to detect moving obstacles and support indoor localization

## Bio particle swarm optimization-reinforcement learning (BPSO-RL)

### Preliminary knowledge

#### Particle swarm optimization

The optimization algorithm of continuous nonlinear functions inspired by the simplified social model is proposed in Ref.^42^. The main methodologies are related to artificial life and evolutionary computation, and they require little computational memory and speed^42^. The swarm artificial life system includes five principles: proximity, diverse response, quality, stability and adaptability^43^. The modified PSO algorithm is presented in Ref.^44^ to introduce inertia weight, and the *i*th particles are updated as Eqs. (7)–(8).

7 $$\begin{aligned} v_i ^ {t+1}= &   \omega v_i^t + c_1 r_1 (pbest_i^t-x_i^t) + c_2 r_2 (gbest^t - x_i^t) \end{aligned}$$

8 $$\begin{aligned} x_i^{t+1}= &   x_i^t + v_i ^ {t+1} \end{aligned}$$

where $x_i^t$ represents the position of the *i*th particle in iteration *t*, and $v_i ^ {t+1}$ is the velocity of the particle in the next iteration $t+1$. $\omega$ denotes the inertia weight introduced in Ref.^44^, $c_1, c_2$ are personal and global parameter, and $r_1, r_2$ are the random number within [0, 1]. $pbest_i^t$ indicates the personal best value for the *i*th particle, and $gbest^t$ is the global best value in the swarm.

The PSO algorithm has strong distributed ability and excellent robustness in wide applications with a little modification, and it can easily be hybridized with other algorithms to improve performance^43^. However, the basic PSO algorithm has the drawback that it has a high possibility of falling into the local optimum when solving the combinatorial optimization problem. It may result in many invalid searches, and the balance between local exploitation and global exploration should be paid attention to Refs.^3,43^.

#### Vicsek model

Vicsek et al. presented a dynamics model of self-ordered motion in the system of particles to investigate transport, clustering and migration transition^45^. The Vicsek model is the basic model of the multi-agent systems, including some key features, such as changing neighbourhood, local interaction and dynamic behaviour^46^. The particles were moving continuously on the surface with biologically motivated interaction and moving with a constant absolute velocity. The particles spin in the same direction as their neighbourhood in a square region with biologically motivated interactions^47^. The position and angles of the particles are updated according to Eqs. (9)–(10).

9 $$\begin{aligned} x_{i}^{t+1}= &   x_{i}^{t} + v_{i}^{t}\Delta t \end{aligned}$$

10 $$\begin{aligned} \theta _{i}^{t+1}= &   \left\langle \theta ^{t} \right\rangle _{r} + \Delta \theta \end{aligned}$$

where $x_{i}^{t+1}$ is the position of the *i*th particle in the timeslot $t+1$, and the $v_{i}^{t}$ is the velocity. $\theta _{i}^{t+1}$ denotes the angle of the *i*th particle, which is gained through Eq.(10). $\left\langle \theta ^{t} \right\rangle _{r}$ denotes the average direction of the particles within the circle of the radius *r*, and $\Delta \theta$ is the noise from the interval $[-\frac{\eta }{2},\frac{\eta }{2}]$.

#### Q-learning

The Q-learning (QL) method can adapt the mobile robots’ behaviour in the workspace for path optimization, such as Refs.^48–50^. It enhances the robot for action based on the policy, and the environment returns the states and rewards. The Q-value is updated as Eq. (11), and $Q(s_t, a_t)$ denotes the current Q-value for the action $a_t$ at state $s_t$ at timeslot *t*.

11 $$\begin{aligned} \begin{aligned} Q(s_t, a_t)&= Q(s_t, a_t) + \alpha \left[ r(s_t, a_t) \right. \\&\quad \left. + \gamma \cdot \max _{a \in {\mathscr {A}}} Q(s_{t+1}, a) - Q(s_t, a_t) \right] \end{aligned} \end{aligned}$$

where $\alpha$ represents the learning rate, and $r(s_t, a_t)$ is the reward after taking action $a_t$. $\gamma$ is the discount factor between (0, 1), and $\max _{a \in {\mathscr {A}}} Q(s_{t+1}, a)$ represents the maximum Q-value for the next state $s_{t+1}$.

### BPSO-RL

AGVs operating in the industrial environment usually aim to obtain the optimal path and achieve obstacle avoidance for safe operation. The proposed BPSO-RL approach combines the advantage of the swarm intelligence algorithm and the reinforcement learning approach. The Q-learning method is effective for decision-making, while it would slowly converge in a large state-action space, while the swarm intelligence algorithm is not. The BPSO algorithm minimises the fitness value of the defined objective function, which can quickly explore and exploit the search space to gain the optimal global path.

**Fig. 1:**
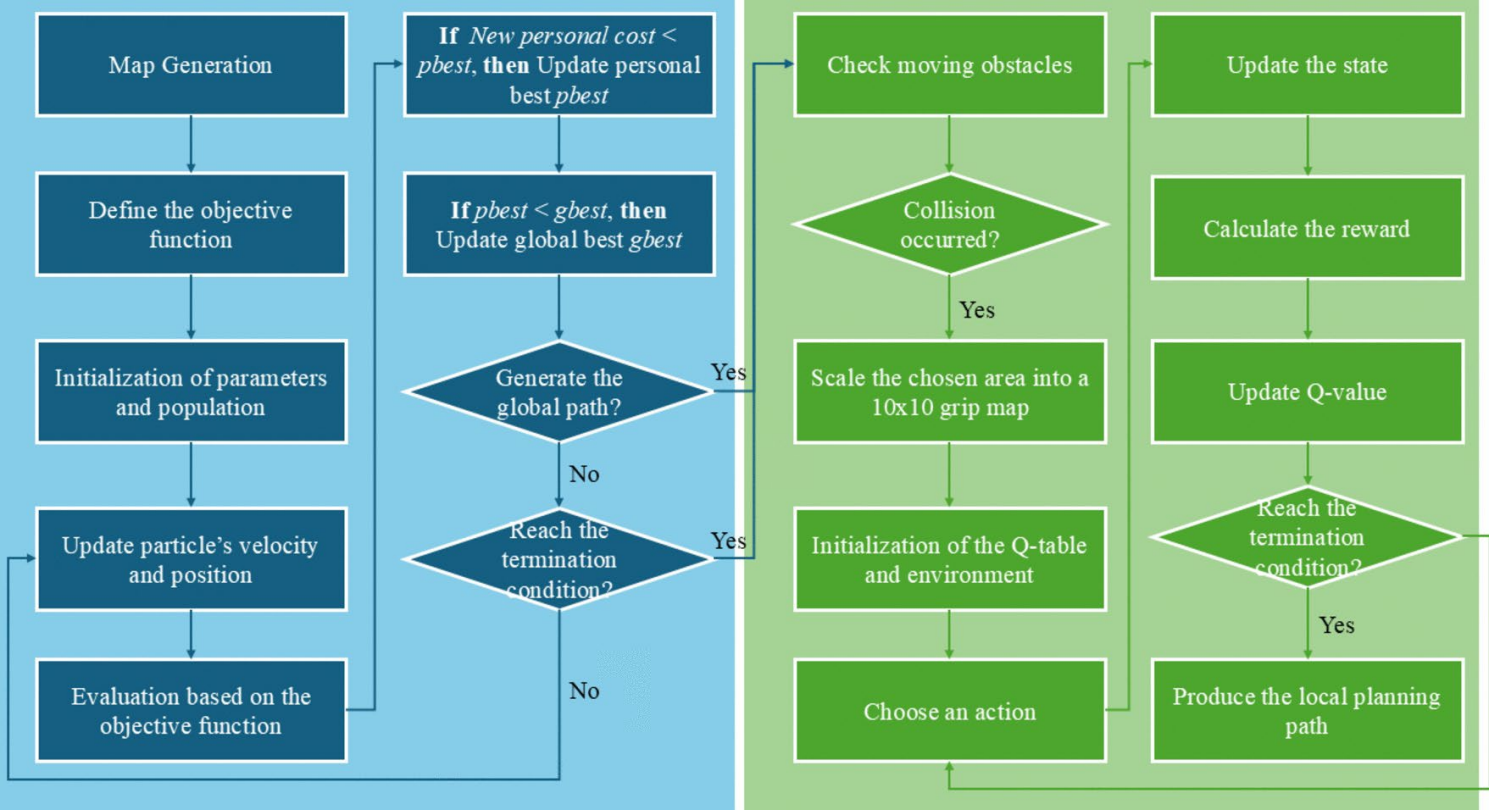
The flowchart of BPSO-RL.

The proposed BPSO-RL approach first supports the global path planning by the proposed BPSO algorithm and then achieves local path planning by the QL method. For the AGV path planning, the flowchart of the BPRO-RL algorithm is illustrated in Fig. 1, where the blue area represents the global path planning, and the green area represents the local path planning. The approach generates the storage map in the industrial environments and defines the objective function as Sect. Problem statement.

The proposed BPSO algorithm for global path planning is inspired by the PSO algorithm and the biological interaction of the system consisting of the particles, and it modifies the updating equation of the velocity of particles in Algorithm 1. The equation of updating the velocity is Eq. (12), it added the angle as $\theta$, and the time interval is represented by $\Delta t$. The angle $\theta$ is randomly generated at the initialization by $2 \pi (rand - 0.5)$ for each particle. It enhances the searchability of the swarm and improves the convergence speed to generate optimal solutions. Introducing randomly generated angles enhances the particles’ movements to avoid premature convergence to local optima, which accelerates the exploration of the search space and improves the diversity of particles.

**Algorithm 1:**
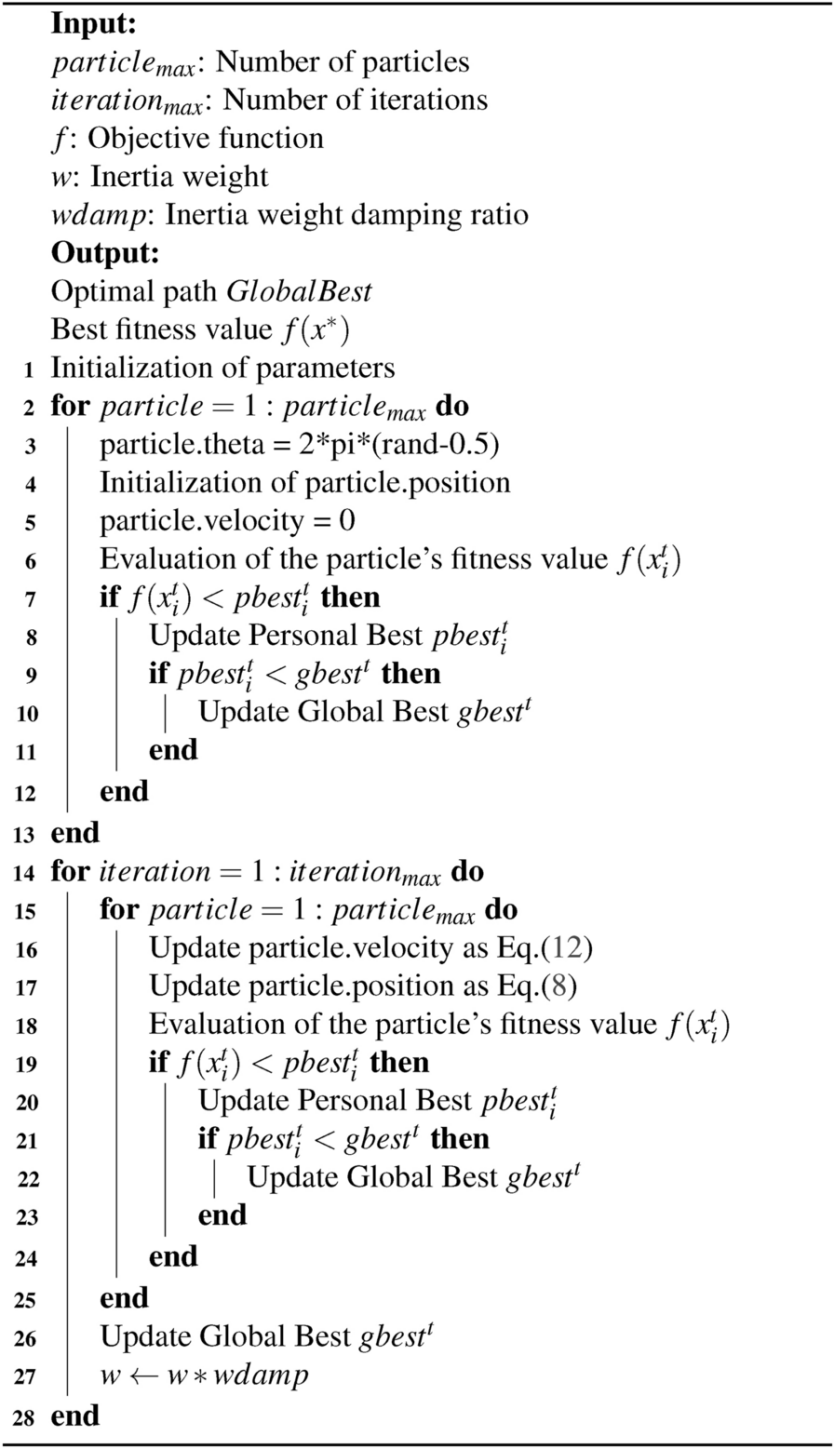
BPSO algorithm

The initialization of the population includes randomly distributed positions and angles $\theta$, and the velocities are set as 0. If the velocities or positions are out of the search space, the particles’ velocities or positions would be limited to the nearest boundary value. Then, the BPSO algorithm updates the particle’s velocity and position and then evaluates the solution based on the objective function. If the new solution’s fitness value is less than the personal best *pbest*, then updating *pbest*. If *pbest* is less than the global best *gbest* of the population, then *gbest* is updated. If the global best value is not updated in 10 iterations, then the generated path is treated as the optimal global path. Otherwise, the global path is generated after the iteration terminates.

12 $$\begin{aligned} v_i ^ {t+1} = \omega v_i^t + c_1 r_1 (pbest_i^t-x_i^t) + c_2 r_2 (gbest^t - x_i^t) + \theta \Delta t \end{aligned}$$

**Fig. 2:**
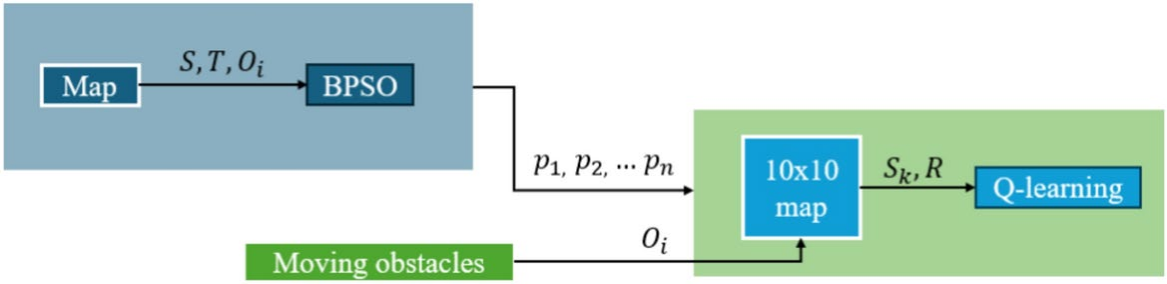
The framework of BPSO-RL.

The BPSO algorithm is used for global path planning in the scenario, and the QL method is used to avoid moving obstacles. The BPSO-RL approach checks for potential collision with moving obstacles. If a moving obstacle appears, the QL method performs the local path planning. The path is re-planned 1 second before the potential collision, and it uses a 10x10 map centred around the original BPSO path for the QL method. The timestamps considered begin at the previous timestamp before the collision occurs and continue for the next ten timestamps. The original BPSO path points within the ten timestamps are scaled into the 10x10 grid map. The method is indicated in Fig. 2. The possible actions include up, down, left and right. If the agent faces an impasse, it will remain stationary. After initialising the environment and the Q-table, the QL method chooses the action as exploration or exploitation. Then, perform the action, update the new state, calculate the reward, and update the Q-value. The local path is obtained if the iteration reaches the termination condition.

## Experiments

### Comparative analysis

The comparative analysis and path planning simulations were conducted on a computer with an Intel Core i5-13600KF processor, the NVIDIA GeForce RTX 4070 GPU, and 32 GB RAM. The proposed BPSO algorithm is compared with other evolutionary algorithms, including the Canonical PSO^44^, Real-coded GA^51^, and Transit Search (TS) algorithms^52^. GA is a popular swarm intelligence algorithm in path planning, and TS is a new swarm intelligence algorithm.

**Fig. 3:**
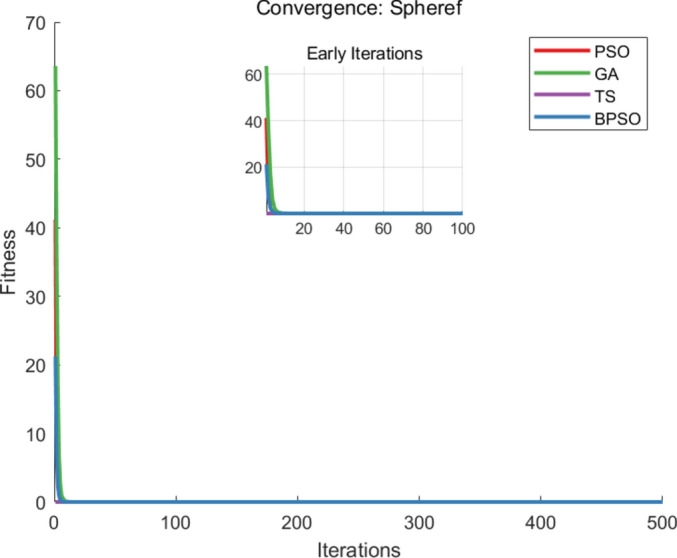
The convergence curve of $F_{1}(x)$.

**Fig. 4:**
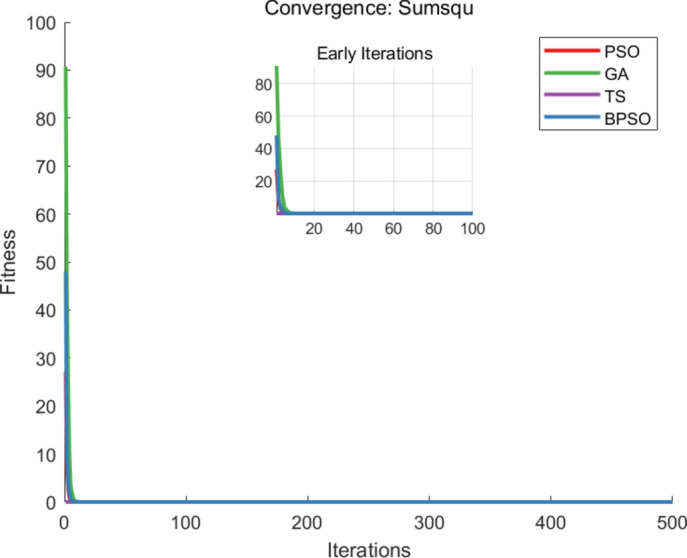
The convergence curve of $F_{2}(x)$.

**Fig. 5:**
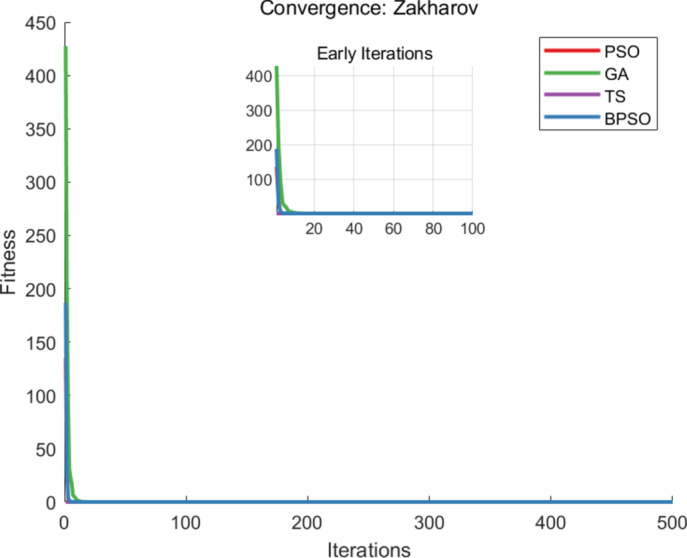
The convergence curve of $F_{3}(x)$.

The benchmark functions are listed in Table 1, and each algorithm runs 20 times, with 500 iterations per execution. For each execution, the parameter settings for each algorithm are shown in Table 2. The metrics of comparisons include the number of iterations, the average runtime when generating the optimal solution, and the accuracy of the solution that is the best fitness value, and the comparison of the best fitness values, iteration and runtime are recorded in Table 3. The convergence curves of the benchmark functions are shown in Figs. 3, 4 and 5.

**Table 1:** Test functions.

Function	Name	Equation	Search Range
$F_1(x)$	Sumsqu	$F_1(x)= \sum \limits _{i=1}^{d} ix_i^2$	[-100,100]
$F_2(x)$	Sphere	$F_2(x)= \sum \limits _{i=1}^{d} x_i^2$	[-100,100]
$F_3(x)$	Zakharov	$F_3 (x)= \sum \limits _{i=1}^{d} x_i^2 + {(\sum \limits _{i=1}^{d} 0.5ix_i^2)}^2+{(\sum \limits _{i=1}^{d} 0.5ix_i^2)}^4$	[-100,100]

**Table 2:** Parameter settings.

Algorithm	Parameter
BPSO	$iter_{max} = 500,~n_{pop} = 100,~w=0.8,~w_{damp}=0.8,~dt=1,~c_{1} = 1.5,~c_{2} = 1.5$
PSO	$iter_{max} = 500,~n_{pop} = 100,~w=0.8,~w_{damp}=0.8,~c_{1} = 1.5,~c_{2} = 1.5$
GA	$iter_{max} = 500,~n_{pop} = 100,~pc=0.7,~gamma=0.4,~pm = 0.3,~mu = 0.1$
TS	$iter_{max} = 500,~n_{pop} = 100,~ns = 2,~SN = 50$

**Table 3:** Mean fitness values, iterations and runtime.

Algorithm		$F_{1}(x)$	$F_{2}(x)$	$F_{3}(x)$	Mean
BPSO	Fitness value	0.0000	0.0000	0.0000	0.0000
	Iteration	300.2	279.7	288.25	289.38
	Runtime	0.2254	0.2099	0.2083	0.2145
PSO	Fitness value	0.0000	0.0000	0.0000	0.0000
	Iteration	342.9	343.45	346.6	344.32
	Runtime	0.2820	0.2817	0.3064	0.2900
GA	Fitness value	0.0000	0.0000	0.0000	0.0000
	Iteration	499.2	498.7	498.2	498.7
	Runtime	0.2411	0.2423	0.2680	0.2471
TS	Fitness value	0.0000	0.0000	0.0000	0.0000
	Iteration	491.05	491.15	490	490.73
	Runtime	0.3636	0.3626	0.3989	0.3750

From the comparative analysis, the proposed BPSO algorithm can get the optimal solution with less iteration and runtime in functions $F_1(x)$, $F_2(x)$, and $F_3(x)$. The iteration is recorded when the algorithm reaches the best fitness value. The runtime is calculated as the product of the average time of each iteration and the average iterations to obtain the optimal solution. The BPSO algorithm saves 15.96%, 41.97%, and 41.03% for iterations and saves 26.03%, 13.19%, and 42.8% computational time, with the comparison with PSO, GA, and TS algorithms, respectively.

The PSO algorithm generates the best solution with fewer iterations than GA and TS algorithms, while GA reaches the global solution with less runtime than PSO and TS algorithms. The TS algorithm performs well for small-scale problems. The presented BPSO algorithm and the PSO algorithm have high solution quality in unimodal problems but may suffer slow convergence in multimodal problems.

### Path planning

The parameter settings for path planning by the BPSO algorithm are the number of population size is 150, the maximum number of iterations is 150, inertia weight *w* is 1, inertia weight damping ratio *wdamp* is 0.98, and the personal and global learning coefficients *c*1 and *c*2 is 1.5, and the time interval *dt* is 1. The parameter settings of the PSO algorithm about the population size, the maximum number of iterations, inertia weight, inertia weight damping ratio, and the personal and global learning coefficients are set in the same way as the BPSO algorithm.

**Fig. 6:**
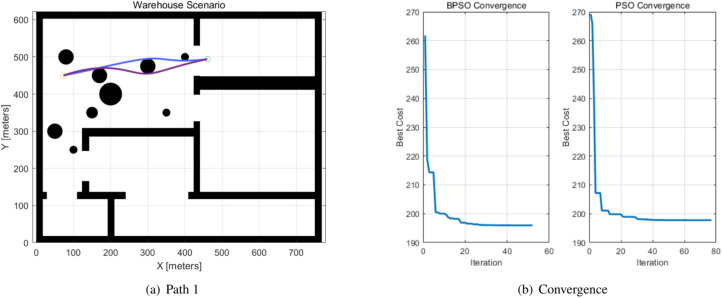
Path 1 generated by the BPSO and PSO algorithms.

**Fig. 7:**
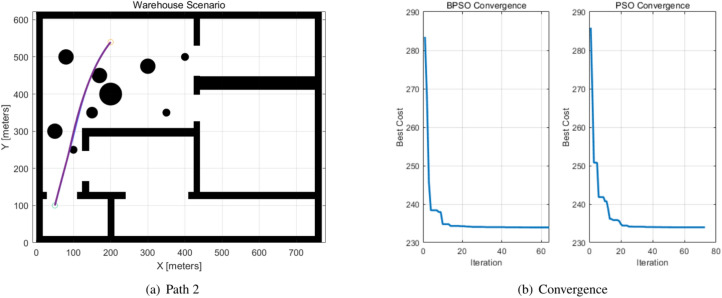
Path 2 generated by the BPSO and PSO algorithms.

The paths generated by the BPSO and PSO algorithms for global path planning are compared in Table 4, the start and target, path length and iterations, and fitness values are listed, and they are indicated as Path 1 and Path 2. The paths and the convergence of the BPSO and PSO algorithms are shown in Figs. 6, 7, and the BPSO path is indicated by blue, while the PSO path is indicated by purple. The orange circle denotes the start location, and the blue circle denotes the target location. BPSO reduced 22.3981% iterations in two generated paths.

**Table 4:** Path generation.

Paths	Start	Target	Algorithm	Path Length	Iteration	Fitness Value
Path1	(73,450)	(460,494)	BPSO	**391.9610**	**52**	**195.9805**
			PSO	395.5416	77	197.7709
Path2	(200,540)	(50,100)	BPSO	**467.8940**	**64**	**233.9505**
			PSO	467.9600	73	233.9800

Significant values are in bold.

**Fig. 8:**
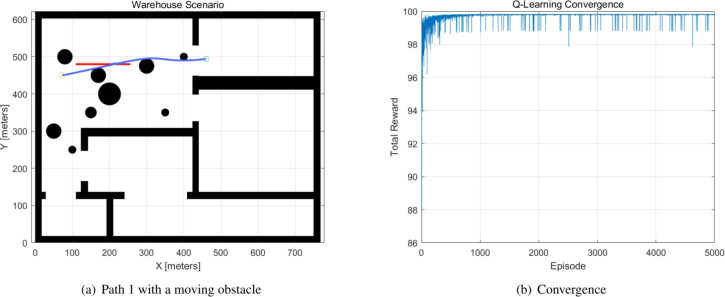
Path 1 and the convergence curve.

**Fig. 9:**
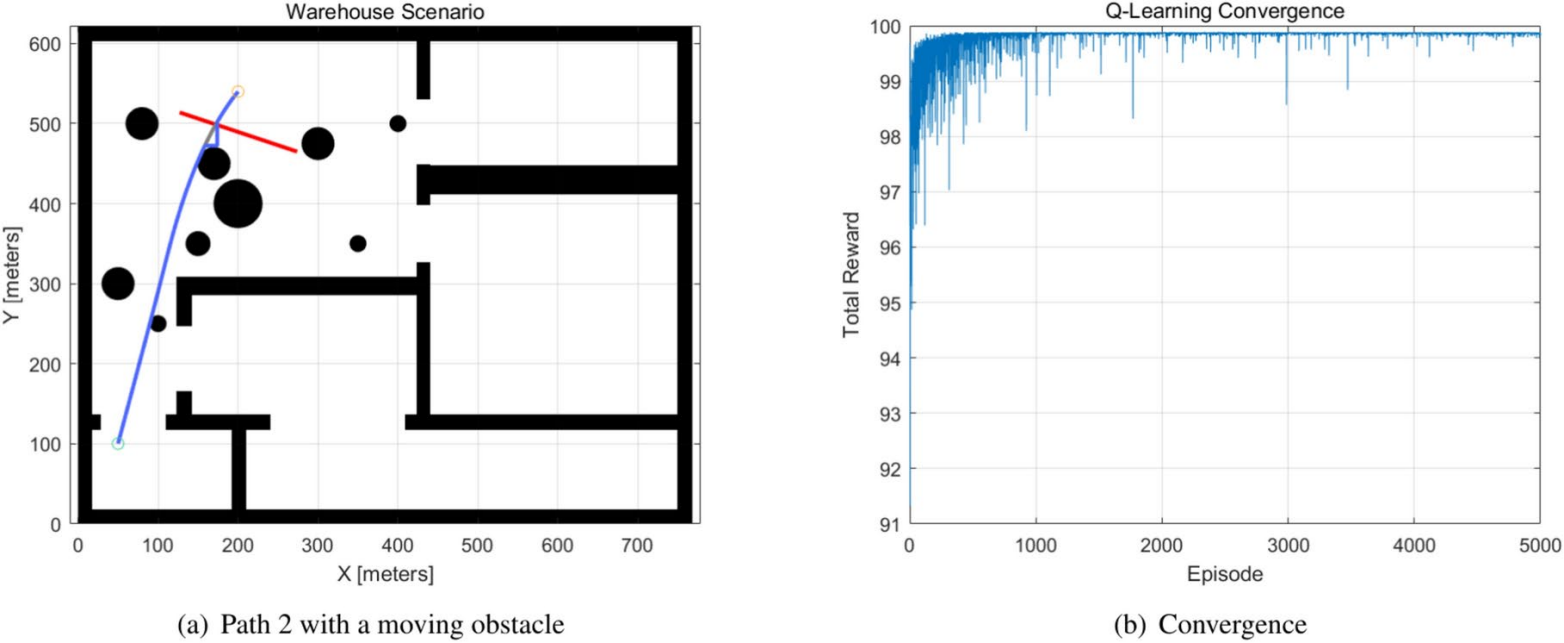
Path 2 and the convergence curve.

The moving obstacles are allocated to the generated path, and the Q-learning method is used to avoid the moving obstacles. The parameters of the moving obstacles are indicated in the Table 5. The moving obstacles 1 and 2 occurred in Path 1 and Path 2. When a moving obstacle appears, the Q-learning method modifies the global path to avoid the moving obstacle. The Q-learning method generates the new path one second before the collision occurs. The parameters of the Q-learning method are $alpha = 0.1, ~gamma = 0.9, ~epsilon = 0.1$, and the number of episodes is 5000, with a 10x10x4 Q-table. The simulation results are demonstrated in Figs. 8, 9. The Q-learning is operated on a 10x10 map, and the figures also show the convergence curve of the total reward.

**Table 5:** Moving obstacles.

Obstacle	Start	Velocity x	Velocity y	Collided time	Path
1	(106,480)	3	0	$t=35$	BPSO Path 1
2	(124,515)	3	-1	$t=16$	BPSO Path 2
3	(127,402)	2	2	$t=12$	BPSO Path 3
4	(210,260)	0	5	$t=28$	BPSO Path 3

**Fig. 10:**
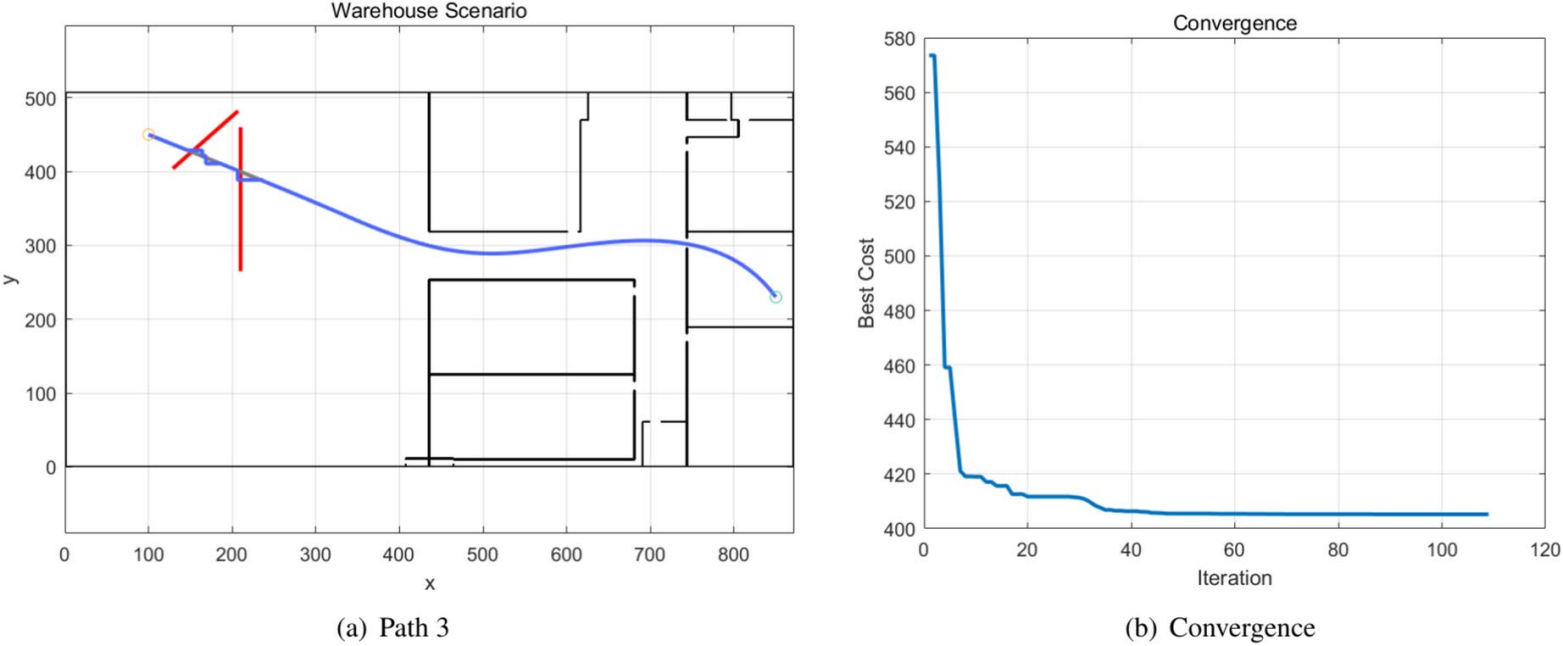
Path 3 generated by the BPSO algorithm.

**Fig. 11:**
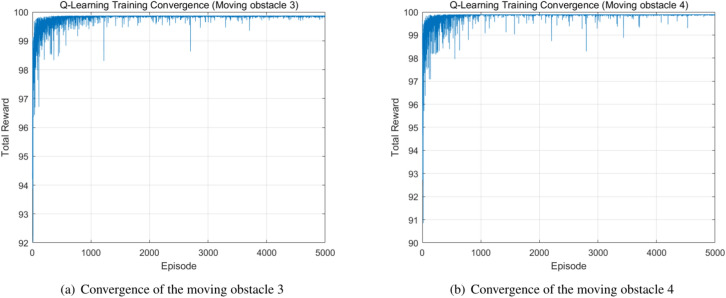
The Q-learning convergences of the moving obstacles 3 and 4.

The moving obstacles 3 and 4 appeared in Path 3, which is a different scenario extracted from a real industrial warehouse. The BPSO algorithm generates the global path as Path 3, which is demonstrated in Fig. 10, and the convergence of the BPSO algorithm is also shown in the figure. The paths of the moving obstacles 3 and 4 are shown in red, and Path 3 is indicated by the blue path. The Q-learning method performs local path planning and modifies the global path. The original BPSO path is highlighted in grey, and the new path generated by the Q-learning method is indicated in blue and connected to the original path. The Q-learning convergences of the moving obstacles 3 and 4 are shown in Fig. 11.

## Conclusion

Path planning is treated as the NP-hard problem, as the exponential growth of the solution space makes it impossible to generate the optimal path in polynomial time. AGV path planning requires generating a safe path in the industrial environment that is modelled as the 2D grid map, with the specific source and target location. The objective function considers the path length and collision avoidance with the weight factors, and it is aimed at minimizing the objective function. This paper presents an improved swarm intelligence algorithm based on the PSO algorithm and inspired by the Vicsek model to improve searchability and performance. The proposed BPSO algorithm modifies the updating equation of the particles’ velocity. It provides a near-optimal solution for AGV path planning and integrates the QL method as the BPSO-RL approach enhances the ability to deal with dynamic scenarios. The reward function considers the collision penalty and the reward of moving and reaching the goal. The BPSO-RL produces the optimal solution with fast computational speed to address the complex path planning optimization problem.

In the comparative analysis, the BPSO algorithm saved 33.99% iterations and 27.34% computational time on average than other algorithms. When generating the paths, the BPSO algorithm reduced 22.40% iterations than the PSO algorithm. The potential impact of the presented BPSO-RL algorithm includes its support for adaptive path planning to enable efficiency and adaptability, which could be used for autonomous navigation systems and smart manufacturing. It has the potential to utilize the decision-making strategy for multi-robot systems or to solve combinational optimization problems, such as network optimization or resource allocation. The real-world applications of the proposed algorithm include AGV’s operation in warehouses that involve unpredictable obstacles, the delivery in logistics and transportation with suitably defined objective functions, or the service robots’ operation. Optimized paths can save operational costs and prevent people or other AGVs from being in a dynamic environment.

However, the limitation of the presented BPSO-RL algorithm is related to possible latency issues in a large-scale environment, and the Q-learning method could not guarantee the completeness of the path generation. Future work is focused on implementing the proposed algorithm in practical application. When integrating the presented algorithm into the sensors and hardware, it would face noisy sensor data. Implementing the neural network or nonlinear filters can deal with the sensor data. Also, the multi-objective optimization algorithm could be used to consider more factors when performing global path planning. The extension of the algorithm from a single robot to a multi-robot system is possible. In a more complex environment, deep reinforcement learning provides the possibility to learn more generalized policies in a large state-action space, such as Proximal Policy Optimization or Deep Q-Netowrks.

## Data Availability

The datasets used and/or analysed during the current study are available from the corresponding author on reasonable request.
